# Maternal vaccinations coverage and reasons for non-compliance - a cross-sectional observational study

**DOI:** 10.1186/s12884-020-03243-w

**Published:** 2020-09-16

**Authors:** David Drezner, Michal Youngster, Hodaya Klainer, Ilan Youngster

**Affiliations:** 1 Sackler School of Medicine, Tel Aviv University, Tel Aviv, Israel; 2 Obstetrics and Gynecology Unit, Shamir Medical Center, Zerifin, Israel; 3 Paediatric Infectious Diseases Unit and the Center for Microbiome Research, Shamir Medical Center, Zerifin, Israel

**Keywords:** Pertussis, Influenza, Vaccine, Compliance, Pregnancy

## Abstract

**Background:**

Maternal influenza and pertussis vaccinations have been proven safe and effective in reducing maternal and infant morbidity and mortality. Though recommended, not all pregnant women receive these important vaccines. We aimed to evaluate the vaccine coverage of maternal immunization in pregnancy for seasonal influenza and acellular pertussis and elucidate the reasons for non-vaccination among pregnant women. The secondary objective was to describe factors that affect vaccine uptake.

**Methods:**

A cross sectional observational study using anonymous questionnaires distributed to women in the maternity ward or pregnant women hospitalized in the high-risk ward, between Nov 2017 and June 2018, In an Israeli tertiary hospital.

**Results:**

Of 321 women approached, 313 were eligible, with a total of 290 women completing the questionnaire (92.6%). We found a 75.9% (95% CI 71–81) and 34.5% (95% CI 29–40) vaccination rate for pertussis and influenza vaccines, respectively. The most prominent reason for not receiving the pertussis vaccine was being under-informed (24%). Influenza vaccine was not received mainly due to concerns about vaccine efficacy (28%). Other factors influencing vaccine uptake included education, prior childbirth and vaccine recommendations made by the provider.

**Conclusion:**

Although maternal vaccination of pertussis and influenza is officially recommended, vaccine uptake is suboptimal. Our study suggests a central role for medical providers in diminishing the concerns about safety and efficacy, and presents novel factors influencing compliance rates, like seasonality and number of prior births.

## Background

*Bordetella Pertussis* and Influenza are causes of potentially lethal, but preventable, respiratory infections in infants and pregnant women [1–3].

Infant vaccination programs, implemented worldwide, are extremely effective in reducing infection, morbidity and mortality for numerous pathogens. Like many countries, Israel has initiated a vaccination program against pertussis and influenza for children. In Israel, pertussis vaccines are administered from the age of 2 months, but protection is acquired only after receiving the third dose at the age of 6 months. Influenza vaccines are recommended from the age of 6 months, leaving young infants vulnerable to both pathogens during the first 6 months of life.

Maternal immunization for pertussis and influenza are a safe and effective way to reduce both maternal and infant morbidity and mortality [4–6]. Infant immunization is achieved by increasing maternal immunity therefore preventing vertical transmission, and by the transfer of protective immunoglobulins to the fetus via the placenta [7]. In the UK, following the 2012 pertussis epidemic, maternal vaccinations were administered resulting in a 78% reduction in pertussis cases and a 68% reduction in hospital admissions in infants younger than 3 months of age during the first 9 months of 2013 compared with the same period in 2012 [8]. As for influenza, pregnant women are more likely to experience severe complications compared with the general population [9]. Maternal infection is also hazardous to the fetus and may cause serious morbidities such as preterm birth, fetal death and even possible fetal malformations [10]. Maternal vaccinations are effective in preventing maternal infection [11] and reduce infant morbidity if infected [12].

In 2015, Israeli health authorities introduced a recommendation for maternal pertussis vaccine during gestational weeks 27–36, in addition to the influenza vaccine recommendation for pregnant women that are in their second or third trimester during the influenza season (November – March). The vaccines are provided, at no cost to the patients, through the primary care physician or the treating OBGYN clinic. Despite its proven efficacy and safety, and ease of attainment, not all women receive these vital vaccines during pregnancy [13]. In order to improve uptake, it is critical to understand the reasons for non-compliance. Focused interventions targeting factors contributing to low vaccine coverage have been shown to significantly improve vaccination rates [14]. In this study we aimed to estimate the actual national vaccination coverage, and to uncover the main hindrances for receiving maternal vaccines, helping the development of focused strategies for increasing vaccine uptake.

## Methods

A cross sectional observational study was performed. A questionnaire (Supplement 1) was composed based on the possible causes for not receiving vaccinations, as described in previous studies [15, 16]. Financial barriers as a potential cause of noncompliance were emitted from our survey due to the fact that these vaccines are administered at no personal cost in Israel. Demographic details included age, prior number of children and their vaccination status, education, religious grouping and health maintenance organization (HMO) membership. The questionnaire included inquiries about vaccination status, recommendations received and personal opinions.

The questionnaire was handed out personally by an investigator (DD) to consecutive women above the age of 18 years in the Maternity ward and High-Risk ward of the Shamir Medical Center, Israel, on random days, between December 2017 and July 2018, a period that includes the peak influenza season. At the maternity ward participants were required to be within 4 weeks of delivery to minimize recall bias. They were at least 28 weeks pregnant at the time of delivery and were in their second or third trimester of pregnancy during the influenza season, when administration of this vaccine during pregnancy is indicated. Women in the high-risk ward were included if they were at least 37 weeks pregnant, for them to have had sufficient time to be vaccinated. Verbal informed consent was obtained during initial encounter and confirmed by questionnaire retrieval.

### Statistical analysis

All data was stored in a Microsoft Excel spreadsheet on a secured hospital network. The rate of coverage for each vaccine was estimated with 95% confidence interval for proportions. Continuous variables were compared between vaccinated women and non-vaccinated women using independent t-test. The categorical variables were compared using chi-square. A logistic regression model was applied to identify the significant factors contributing to vaccination uptake separately for Influenza & pertussis. Significance level was defined as *p* = 0.05. Analyses were carried out using. IBM SPSS Statistics for Windows, Version 25.0. Armonk, NY: IBM Corp.

### Ethical approval

Shamir Medical Center Institutional Review Board approval was received on November 23, 2017.

## Results

Of 321 women that were approached, 313 met the inclusion criteria (7 women had inadequate language fluency and 1 woman was a foreign citizen that received her healthcare abroad) and 290 completed the questionnaire (92.7% completion rate). Four questionnaires had missing data concerning influenza vaccine uptake and 5 were missing demographic details. Characteristics of the study population are shown in Table 1.

**Table 1:** Select characteristics of Pertussis and influenza virus vaccines uptake in late pregnancy or post-partum women, winter-spring 2017–2018, Shamir Medical Center, Israel

	Pertussis			Influenza		
	N.	Vaccinated %	***P*** value	N.	Vaccinated %	***P*** value
**Total**	**290 (100%)**	**75.9**		**286 (100%)**	**34.5**	
**Ward**						
Maternity	272 (93.8)	75.7	*0.854*	268 (93.7)	35.1	*0.881*
High Risk	18 (6.2)	77.8		18 (6.3)	33.3	
**Age (19–44)**						
18–24	37 (13.1)	64.9	*0.237*	35 (12.5)	28.6	*0.241*
25–34	183 (64.7)	76.5		182 (65.2)	39.0	
35–44	63 (22.3)	79.4		62 (22.2)	29.0	
**Prior children (0–7)**						
0	79 (28.0)	86.1^c^	*0.001*	78 (28.1)	48.7	*0.034*
1	96 (34.0)	79.2^c^		96 (34.5)	32.3	
2	59 (20.9)	74.6		58 (20.9)	31.0	
3+	48 (17.1)	54.2^b^		46 (16.5)	26.1	
**Prior children vaccinated**						
Yes	178 (91.8)	75.8	*0.006*	175 (91.6)	33.7	*0.023*
No/partial	16 (8.2)	43.8		16 (8.4)	6.3	
**Education**						
No degree	136 (48.1)	66.9	*0.001*	133 (47.7)	26.3	*0.002*
≥ First degree	147 (51.9)	84.4		146 (52.3)	43.8	
**Religious group**						
Jewish	231 (81.9)	79.2^c^	*0.013*	228 (82.0)	37.3	*0.25*
Jewish ultra-orthodox	17 (6.0)	52.9^b^		17 (6.1)	17.6	
Other religion^a^	34 (12.1)	64.7		33 (11.9)	33.3	
**Health care provider**						
Clalit	149 (52.3)	71.8^c^	*0.009*	147 (52.3)	30.6	*0.174*
Maccabi	91 (31.9)	86.8^b^		90 (32.0)	42.2	
Others	45 (15.8)	66.7^c^		44 (15.7)	38.6	
*Meuchedet*	28 (9.8)	71.4		28 (10.0)	57.1	
*Leumit*	13 (4.6)	46.2		13 (4.6)	7.7	
*IDF*	4 (1.4)	100.0		3 (1.1)	0	
**Vaccine recommended**						
Yes	272 (93.8)	80.5	*< 0.01*	221 (77.3)	43.9	*< 0.01*
No	18 (6.2)	5.6		65 (22.7)	4.6	
**If so, who recommended**						
Obstetrician only	152 (56.9)	83.6	*0.22*	95 (43.8)	44.2	*0.941*
Obstetrician and others	68 (25.5)	73.5		57 (26.3)	43.9	
Others only	47 (17.6)	80.9		65 (35.0)	41.5	
**Season of delivery/late pregnancy**						
Nov-March	105 (36.8)	82.9	*0.033*	103 (36.7)	47.6	*< 0.001*
April–July	180 (63.2)	71.7		178 (63.3)	27.0	
**Folic acid uptake**						
Yes	258 (92.1)	78.3	0.003	254 (92.0)	36.6	0.192
No	22 (7.9)	50.0		22 (8.0)	27.7	
**Prior influenza vaccination (not during pregnancy)**						
Yes	92 (33.3)	85.9	0.009	92 (33.6)	57.6	< 0.01
No	184 (66.7)	71.7		182 (66.4)	22.5	

^a^other religions: Christian, Muslim, Bedoin or ‘others’

^b^Reference group in categories with 3 or more subgroups

^c^Statistically significant difference versus reference group within subgroup

**Fig. 1:**
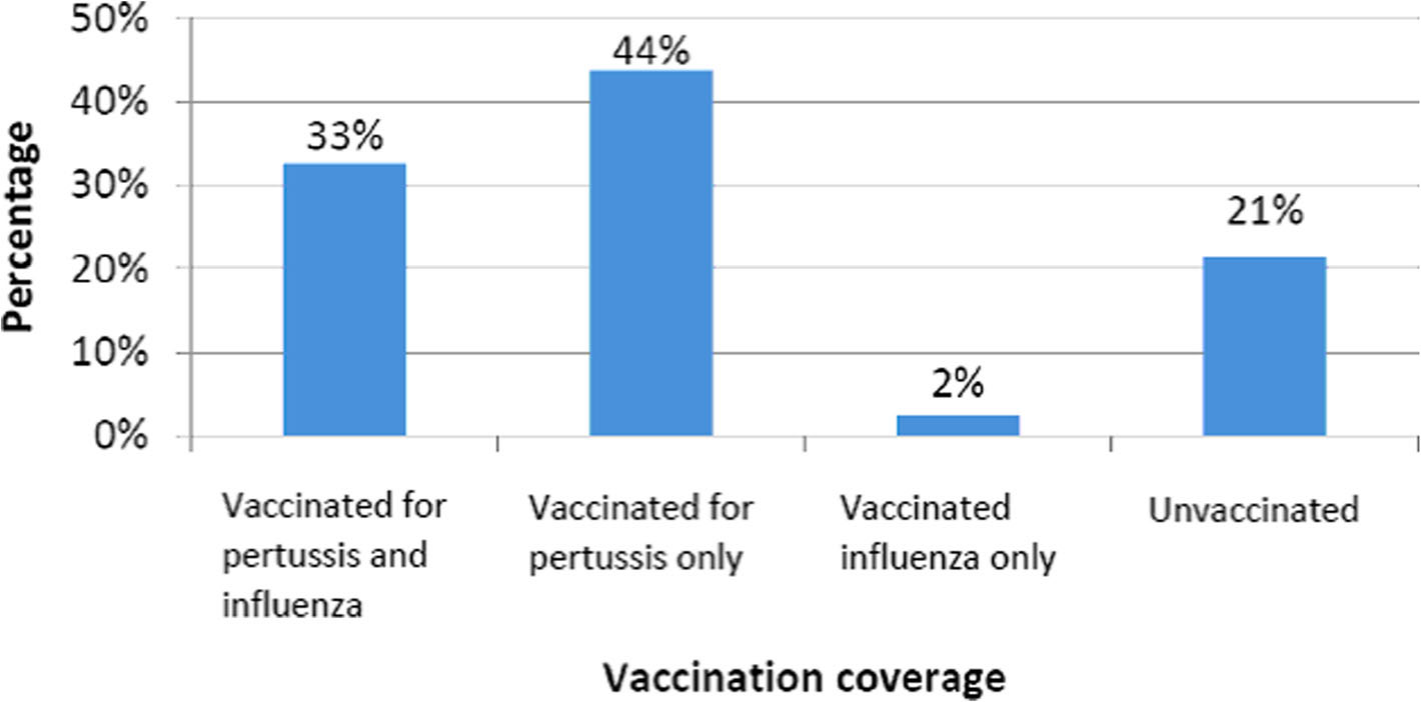
Pertussis and influenza virus vaccines uptake (*n* = 286) in late pregnancy or post-partum, winter-spring 2017–2018, Shamir Medical Center, Israel

Seventy six percent (95% CI 71–81) and 34.5% (95% CI 29–40) of participants reported being vaccinated for pertussis and influenza respectively (Table 1). Of the 286 women who reported vaccination status for both pertussis and influenza only 33% reported receiving both vaccines while 21% received neither (Fig. 1).

**Fig. 2:**
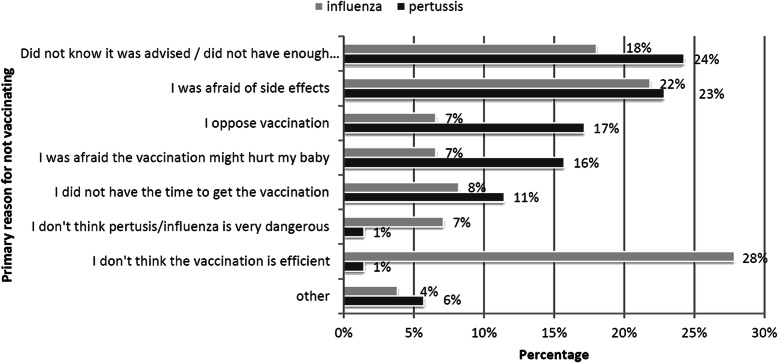
Primary reasons for not vaccinating against pertussis (*n* = 70) and influenza (*n* = 183) reported by survey population

Figure 2 shows the primary reasons reported by responders for not receiving the recommended vaccines during pregnancy. The most common reason for not vaccinating against pertussis was being uninformed of vaccination recommendations or lack of information about the vaccine (24%). Influenza vaccine was not received mainly due to concerns about poor vaccine efficacy (28%). Both were followed by concerns about side effects, (23 and 22% respectively).

The mean age of respondents at the time of the survey was 30.5 years (19–44, SD ±5.2). Age group did not significantly impact vaccine uptake. Gestational week at delivery/hospitalization averaged 38.9 (28.0–42.0, SD ±1.8) weeks for both groups and no statistically significant difference was found between vaccinated and non- vaccinated groups.

Table 1 presents the vaccination coverage according to demographic characteristics. Having three or more prior children or having non-vaccinated children at home was associated with lower vaccination uptake. Women defining themselves as Jewish ultra-orthodox had lower vaccination rates compared to other groups. Academic education had a positive effect on vaccination status, as did receiving a recommendation from a health care worker, irrespective of the role of the recommending person. Regarding Health Maintenance Organization (HMO) influence, significantly higher pertussis vaccine coverage was reported by those who belonged to Maccabi Healthcare compared to the other groups. As for influenza, a similar trend was observed but did not reach statistical significance.

**Fig. 3:**
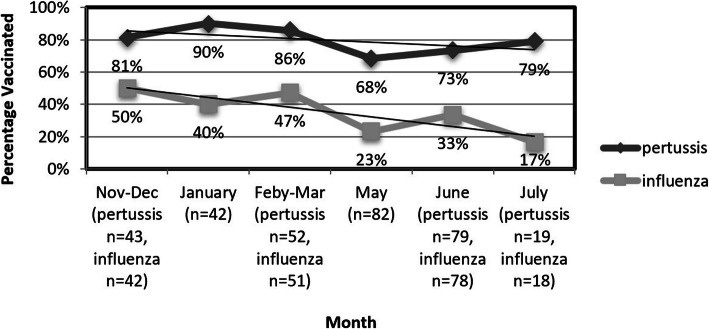
Pertussis and influenza vaccines coverage by month of delivery or late pregnancy (in High Risk ward)

Season of birth significantly affected vaccination rates, influencing influenza uptake more than pertussis. Women in late pregnancy (> 37 weeks) or those who delivered during the influenza season (Nov-March) were more likely to be vaccinated compared to those who reached the end of their pregnancy during spring months (April–July) even though indications to be vaccinated applied to all women in this study. Figure 3 shows the distribution of vaccination rates by month of delivery.

A multivariable analysis was conducted to account for cross-interactions between demographic and behavioral variables. Table 2 presents the odds ratio when considering multiple variables. Noticeably, for pertussis the most influencing factors were fewer prior children, followed by the age group 35–44 which tended to vaccinate more than the younger age group. Folic acid uptake during pregnancy (possibly as a general marker of compliance) indicated higher uptake as well. As for influenza, only educational status and winter season birth correlated significantly with higher vaccine acceptance. These factors were not found to be statistically significant in the pertussis uptake analysis.

**Table 2:** Odds Ratio and 95% confidence interval (CI) for multivariable analysis applying to variables influencing vaccine uptake in pregnant women

	Pertussis		Influenza	
	OR (95% CI)	***P*** value	OR (95% CI)	***P*** value
**Age group**				
18–24	1.00		1.00	
25–34	2.26 (0.88–5.83)	*0.091*	1.22 (0.48–3.06)	*0.676*
35–44	4.60 (1.38–15.37)	*0.013*	0.80 (0.26–2.50)	*0.705*
**Prior children**				
0	7.36 (2.38–22.73)	*0.001*	1.84 (0.67–5.04)	*0.236*
1	3.34 (1.28–8.69)	*0.013*	0.88 (0.33–2.33)	*0.795*
2	2.01 (0.78–5.16)	*0.147*	0.85 (0.31–2.35)	*0.750*
3+	1.00		1.00	
**Education**				
No degree	1.00		1.00	
≥ First degree	1.83 (0.94–3.57)	*0.078*	1.98 (1.09–3.58)	*0.024*
**Religious group**				
Jewish	1.00		1.00	
Jewish ultra-orthodox	0.61 (0.19–1.94)	*0.404*	0.46 (0.12–1.87)	*0.280*
Other religion^a^	1.05 (0.41–2.70)	*0.922*	1.45 (0.60–3.55)	*0.411*
**Health care provider**				
Clalit	1.00		1.00	
Maccabi	2.26 (0.98–5.18)	*0.055*	1.62 (0.87–3.03)	*0.130*
Others	0.59 (0.25–1.38)	*0.222*	1.17 (0.52–2.63)	*0.708*
**Season of delivery/late pregnancy**				
Nov-March	1.98 (0.98–3.99)	*0.058*	2.58 (1.47–4.53)	*0.001*
April–July	1.00		1.00	
**Folic acid uptake**				
Yes	3.15 (1.09–9.09)	0.034	1.68 (0.55–5.15)	0.361
No	1.00		1.00	

^a^other religions: Christian, Muslim, Bedoin or ‘others’

During the course of the study a question regarding exposure to social media was added to the questionnaire. One hundred and two out of 182 women (56%) stated that they had been exposed to discussions regarding vaccinations on different social media platforms. 37.3% of those exposed (*n* = 38) admitted that these discussions had an influence on their decision to receive or not receive these vaccinations.

## Discussion

We describe a maternal vaccination rate of 75.9% for pertussis and 34.5% for influenza. This maternal pertussis vaccination coverage in Israel is comparable to that reported in other countries (for example: England − 70-75% [17], United States - 54.4% [13]) but still falls short of national target rates aiming for universal coverage of > 90%. Influenza coverage, on the other hand, is far from satisfactory even when compared to coverage in other countries like the United States (49.1% [13]) or Belgium (45% [18]). This low maternal influenza vaccine uptake is found despite comparable recommendation rates given by primary caregivers, a factor that was found to be a strong predictor of maternal acceptance. In a study published by the Center for Disease Control, 81.0% received recommendation for influenza vaccine resulting in a 49.1% vaccination rate [13], compared to 77.3% who reported receiving a recommendation in our study resulting in only 34.5% vaccination. This implies that providing vaccine recommendations as a stand-alone strategy is not enough. This observation may in part be due to a difference in perception of prevalence or severity of these diseases. These results highlight the need for health authorities to implement strategies to actively increase uptake of these vaccines, with an emphasis on influenza.

We report several reasons for declining maternal vaccinations. Our results point out that 24% of pregnant women did not receive the pertussis vaccine primarily because they were uninformed about vaccine recommendations and expected benefits, information that could have been provided by practitioners. In Israel, both vaccines can be obtained either through the primary care physician or the OBGYN clinic, leaving ample opportunity to inform pregnant women. Moreover, concerns about safety played a role in reducing uptake in 39% of women who did not receive the vaccine, including concerns about side effects to themselves and potential harm to their fetus. As for influenza, the reasons were quite different as 28% declined the vaccine due to the belief that the influenza vaccine is ineffective and only 18% because they were uninformed about vaccination recommendations. Safety concerns were still an issue as 29% deferred for these reasons. Emphasis of vaccine recommendations is apparently crucial and should be an important first step in any program to increase vaccine uptake in this population. We suggest that the protective effects and overall efficacy and safety should be actively presented to patients. More specifically, the protective effect of both vaccines on the fetus and the newborn baby should be emphasized. These results largely reflect current data from previous studies concluding that knowledge concerning vaccine recommendations, safety and efficacy has a large impact on maternal uptake [12, 15].

Our results highlight multiple determinants influencing vaccine uptake. As previously reported by others, higher education and vaccine recommendations by the provider were predictors of vaccination. Having prior children had an incremental negative effect on vaccine uptake, i.e. - the more children at home, the larger the proportion of unvaccinated mothers. These findings may be an indirect reflection of economic status, a well-known determinant not directly assessed in this study. An alternative explanation may be the fact that these vaccines are a relatively recent addition to the health recommendations in pregnancy, and mothers who have been through multiple pregnancies may be uninformed, relying on previous experience to decide what procedures are necessary. This emphasizes the important role of the healthcare provider, even in highly experienced patients.

The HMO had an impact on vaccine uptake suggesting a difference in organizational procedures to promote vaccinations. Healthcare in Israel is universal and participation in a medical insurance plan through an HMO is compulsory with four public HMOs that every citizen can choose freely to belong to. Our sample largely reflects the national distribution of membership between these providers, albeit Maccabi is slightly over-represented in this study. The smaller providers were merged into a single reference group for statistical measures. Regarding pertussis, ‘Maccabi’ stood out with a remarkable 86.8% coverage. In contrast, Influenza coverage was highest by members of the ‘Meuhedet’ group. This discrepancy may hint that different policies of the healthcare-providing organization can influence vaccine uptake, urging local officials to investigate the differences between HMOs vaccination publicity efforts and increase their efforts to promote uptake of all recommended vaccines.

Lastly, we introduce a new variable affecting vaccine uptake - season of pregnancy/delivery. We found a significant difference in vaccine uptake for both pertussis and influenza, depending on season. Those who were in late pregnancy or gave birth during the influenza season were more likely to vaccinate than those pregnant during the late influenza season. The difference in influenza uptake is not surprising, as women who approach their second or third trimester in late influenza season may be inclined to believe the danger of contracting Influenza has passed. However, we were surprised that the same trend was found in the pertussis vaccine uptake, even though the recommendation is not season dependent. The linkage between the vaccinations should make providers emphasize the importance of the pertussis vaccine during the warm months. Another concern is that many surveys regarding pertussis coverage are collected in parallel to influenza uptake, meaning that studies performed during peak influenza season are potentially causing over-estimations of yearly pertussis uptake.

Our observation regarding exposure to social media and its impact upon vaccine acceptance should be taken into consideration by regulators and policy makers as well. We showed that in one-fifth (20.9%) of women the decision whether to receive the vaccines was influenced by social media. It is essential that we make sure reliable information is spread through these channels. Furthermore, providers need to inquire about their patients’ sources of information and point them towards reliable sources.

The strengths of this study include a > 90% questionnaire completion rate, and the institution in which we conducted this study, that is, a tertiary center with > 9000 yearly deliveries representing all local ethnic and demographic groups. However, as this was a self-reported questionnaire study, limitations include a possible recall bias and social desirability bias which may have caused overestimation of actual coverage. Another limitation was the language barrier potentially causing some selection bias. However, only 7 out of 320 subjects were excluded from the study due to language barriers, having a minimal influence on the results.

## Conclusions

Although maternal vaccination against pertussis and influenza in pregnancy is nationally recommended and is provided free of charge to the Israeli population, local vaccine uptake of pertussis was suboptimal, while influenza uptake was extremely low. The main reasons we found for non-compliance are consistent with previous data and suggest a strong role of the medical staff in recommending and explaining the necessity of maternal vaccinations and diminishing safety and efficacy concerns. These recommendations should be further emphasized in certain populations we found in our study to be more likely to forego vaccinations, such as low level of maternal education, higher number of prior children and ultra-orthodox Jewish populations. Moreover, the season of pregnancy is significant as we found a trend suggesting lower vaccination rates in women giving birth or being in late pregnancy towards the end of the influenza season. Further studies are needed to validate our results. Evaluation of specific intervention programs directed at non-compliant populations may be a valuable resource in planning nationwide strategies for improving vaccination uptake.

## Supplementary information

**Additional file 1.** Supplementary 1 (Questionnaire) – English translation of data collection questionnaire.

## Data Availability

The datasets used and/or analyzed during the current study are available from the corresponding author on reasonable request.

## Abbreviation

HMO: Health maintenance organization
