# Supplementary material for: Maternal vaccinations coverage and reasons for non-compliance - a cross-sectional observational study
Source: BMC Pregnancy Childbirth. 2020 Sep 16;20:541. doi: 10.1186/s12884-020-03243-w (PMC7493363; doi:10.1186/s12884-020-03243-w)
Supplement: Supplementary file 1 — Additional file 1. Supplementary 1 (Questionnaire) – English translation of data collection questionnaire. [file 12884_2020_3243_MOESM1_ESM.pdf]

Maternal vaccination questionnaire

Initials: ________

Date: ________

Place:

1. Maternity ward (Date of birth: ________)
2. High Risk (Pregnancy week, day ________)

Ethnicity:

1. Secular Jewish
2. Orthodox Jewish
3. Muslim
4. Other ________

Education:

1. No high school
2. High school
3. Professional degree
4. Bachelor's degree
5. Master's degree
6. Doctorate

Health maintenance organization:

1. Clalit
2. Maccabi
3. Meuhedet
4. Leumit
5. IDF

No. of Previous Pregnancies: ________

No. of children (not including current delivery): ________

If you have children, did they get their childhood vaccines?

1. Yes
2. Partially
3. No

Did you get your routine vaccines?

1. Yes
2. Partially
3. No (reason: ____)

Regarding your current pregnancy:

Did you take folic acid supplementation in early pregnancy?

1. Yes

2. No

Did you get the influenza vaccine?

1. Yes (when? ________)
2. No

If not, what was the reason for not getting the influenza vaccine?

1. I did not know it was advised / did not have enough information
2. I was afraid of side effects
3. I was afraid the vaccine might hurt my baby
4. I did not have the time to get the vaccination
5. I oppose vaccination during pregnancy
6. I oppose vaccination in general
7. I don't think influenza is very dangerous
8. I don't think the vaccine is efficient
9. Other: ____
   ____

Did you know about the recommendation to get the influenza vaccine during pregnancy?

1. Yes
2. No

Did your healthcare provider discuss the recommendation with you?

1. Yes
2. No

If yes, which healthcare provider recommended the vaccine?

1. OBGYN
2. GP
3. Pediatrician
4. Midwife/doula
5. Nurse
6. Other _____

Have you been exposed to discussions on social media regarding vaccine safety and efficacy during pregnancy?

1. Yes (which social media platforms? _________)
2. No

Did the social media exposure influence your decision about whether to vaccinate?

1. Yes
2. No
   Please describe the influence: ____

Did you get the pertussis vaccine?

1. Yes (when? ________)
2. No

If not, what was the reason for not getting the pertusis vaccine?

1. I did not know it was advised / did not have enough information
2. I was afraid of side effects
3. I was afraid the vaccine might hurt my baby
4. I did not have the time to get the vaccination
5. I oppose vaccination during pregnancy
6. I oppose vaccination in general
7. I don't think influenza is very dangerous
8. I don't think the vaccine is efficient
9. Other: ____
   ____

Did you know about the recommendation to get the pertussis vaccine during pregnancy?

1. Yes
2. No

Did your healthcare provider discuss the recommendation with you?

1. Yes
2. No

If yes, which healthcare provider recommended the vaccine?

1. OBGYN
2. GP
3. Pediatrician
4. Midwife/doula
5. Nurse
6. Other _____

Have you been exposed to discussions on social media regarding vaccine safety and efficacy during pregnancy?

3. Yes (which social media platforms? _________)
4. No

Did the social media exposure influence your decision about whether to vaccinate?

3. Yes
4. No
   Please describe the influence: ____
